# Flavan-3-ols and Proanthocyanidins in Japanese, Bohemian and Giant Knotweed

**DOI:** 10.3390/plants10020402

**Published:** 2021-02-20

**Authors:** Maja Bensa, Vesna Glavnik, Irena Vovk

**Affiliations:** 1 Laboratory for Food Chemistry, National Institute of Chemistry, Hajdrihova 19, SI-1000 Ljubljana, Slovenia; maja.bensa@gmail.com; 2 Faculty of Chemistry and Chemical Technology, University of Ljubljana, Večna pot 113, SI-1000 Ljubljana, Slovenia

**Keywords:** *Reynoutria*, Polygonum, Polygonaceae, flavanols, catechins, procyanidins, condensed tannins, HPTLC–MS, chemical profiling, fingerprints

## Abstract

Flavan-3-ols and proanthocyanidins of invasive alien plants Japanese knotweed (*Fallopia japonica* Houtt.), giant knotweed (*Fallopia sachalinensis* F. Schmidt) and Bohemian knotweed (*Fal* *lo* *pia* × *bohemica* (Chrtek & Chrtkova) J.P. Bailey) were investigated using high performance thin-layer chromatography (HPTLC) coupled to densitometry, image analysis and mass spectrometry (HPTLC–MS/MS). (+)-Catechin, (−)-epicatechin, (−)-epicatechin gallate and procyanidin B2 were found in rhizomes of these three species, and for the first time in Bohemian knotweed. (−)-Epicatechin gallate, procyanidin B1, procyanidin B2 and procyanidin C1 were found in giant knotweed rhizomes for the first time. Rhizomes of Bohemian and giant knotweed have the same chemical profiles of proanthocyanidins with respect to the degree of polymerization and with respect to gallates. Japanese and Bohemian knotweed have equal chromatographic fingerprint profiles with the additional peak not present in giant knotweed. Within the individual species giant knotweed rhizomes and leaves have the most similar fingerprints, while the fingerprints of Japanese and Bohemian knotweed rhizomes have additional peaks not found in leaves. Rhizomes of all three species proved to be a rich source of proanthocyanidins, with the highest content in Japanese and the lowest in Bohemian knotweed (based on the total peak areas). The contents of monomers in Japanese, Bohemian and giant knotweed rhizomes were 2.99 kg/t of dry mass (DM), 1.52 kg/t DM, 2.36 kg/t DM, respectively, while the contents of dimers were 2.81 kg/t DM, 1.09 kg/t DM, 2.17 kg/t DM, respectively. All B-type proanthocyanidins from monomers to decamers (monomers—flavan-3-ols, dimers, trimers, tetramers, pentamers, hexamers, heptamers, octamers, nonamers and decamers) and some of their gallates (monomer gallates, dimer gallates, dimer digallates, trimer gallates, tetramer gallates, pentamer gallates and hexamer gallates) were identified in rhizomes of Bohemian knotweed and giant knotweed. Pentamer gallates, hexamers, hexamer gallates, nonamers and decamers were identified for the first time in this study in Bohemian and giant knotweed rhizomes.

## 1. Introduction

This study examines three perennial herbaceous plants that belong to the genus *Fallopia* and the family *Polygonaceae* and are globally very problematic invasive alien plant species. The studied plants were: (1) Japanese knotweed (*Fallopia japonica* Houtt.; synonyms: *Reynoutria japonica* Houtt., *Polygonum cuspidatum* Sieb. & Zucc., *Polygonum reynoutria* Makino); (2) giant knotweed (*Fallopia sachalinensis* F. Schmidt; synonyms: *Reynoutria sachalinensis* (F. Schmidt) Nakai, *Polygonum sachalinense* F. Schmidt); and (3) Bohemian knotweed (*Fallopia* × *bohemica* (Chrtek & Chrtkova) J.P. Bailey; synonyms: *Reynoutria* × *bohemica* (Chrtek & Chrtkova), *Polygonum* × *bohemicum* (Chrtek & Chrtkova) P.F. Zika & A.L. Jacobson), the interspecific hybrid of Japanese and giant knotweed.

In the 19th century Japanese knotweed was first brought to Europe from Japan. Japanese and giant knotweed quickly spread across Europe due to their popularity as ornamental garden plants [1]. Japanese, giant and Bohemian knotweed are widely spread in Europe and are today considered to be three alien invasive species. Because of the growth and spread of these knotweeds, they have become a global ecological and economical problem (endangering biodiversity and destroying the infrastructure) in Europe, North America, Australia and New Zealand. Japanese knotweed is also included on the list of the 100 most invasive alien species [2,3].

Knotweeds’ invasive nature comes from their ability to grow almost anywhere. They require very little to thrive and therefore they are often found growing on riverbanks, at the edges of forests, construction sites, near roads, railway embankments, abandoned fields and landfills. Seeds or even small pieces of rhizomes or stems can propagate the spread of knotweeds. Several attempts to eradicate these *Fallopia* species in Europe and North America have proven to be unsuccessful, hence another direction was taken to find positive uses. Japanese knotweed has already been used in traditional Chinese medical treatments such as inflammatory diseases, diuretic problems and diarrhea [4]. Japanese knotweed has also been used as a raw material for isolating stilbene *trans*-resveratrol for the production of food supplements. There is ongoing research in different industries (e.g., food, paper) on how to utilize the great untapped potential of *Fallopia* knotweeds as a raw material for various purposes (e.g., a natural herbicide [5], an antioxidant agent [6]). Fewer studies have been done on Bohemian and giant knotweed plant materials. Among the groups of secondary metabolites found in the rhizomes of Japanese [7,8,9,10,11,12,13,14], Bohemian and giant [7,8,12,13,14] knotweed were phenolic acids [7,8], flavonoids [7,8,9,10,12,13,14,15], stilbenes [7,8,9,10,14,15], anthraquinones (emodins) [9,10,11,14,15] and triterpenic acids [7,8]. Emodin, emodin-8-O-glucoside and emodin-8-O-malonyl-glucoside were reported for rhizomes [11,15] and the bark of the rhizomes [15] of Japanese knotweed. Flavan-3-ols and B-type proanthocyanidins from monomers up to decamers (monomers — flavan-3-ols, dimers, trimers, tetramers, pentamers, hexamers, heptamers, octamers, nonamers and decamers) and some of their gallates (monomer gallates, dimer gallate, dimer digallates, trimer gallates, tetramer gallates, pentamer gallates, hexamer gallates) were tentatively identified in Japanese knotweed rhizomes [9,10]. (+)-Catechin, (−)-epicatechin, (−)-epicatechin gallate, procyanidin B1, procyanidin B2, procyanidin B3, proanthocyanidin B dimer gallate were isolated from the rhizomes and bark of the rhizomes of Japanese knotweed [15]. Other studies of rhizomes of Japanese [7,8,12,13,14], Bohemian [12,14] and giant knotweed, reported the findings of the following proanthocyanidins: monomers [7,8,12,13,14], monomer gallates [7,12,13], dimers [7,8,14], dimer gallates, dimer digallates [13,14] trimers, trimer gallates, trimer digallates, tetramer, tetramer gallates, pentamers, heptamers and octamers [14]. There is no report about quantifications of the total content of monomers and dimers of proanthocyanidins in rhizomes of Japanese, Bohemian and giant knotweed.

The aim of our work was to: (1) obtain chromatographic fingerprints for rhizomes of Japanese, Bohemian and giant knotweed; (2) compare chromatographic fingerprints for rhizomes and leaves of the same knotweed species; (3) compare the total contents of flavan-3-ol (monomers) and dimers in all three knotweeds; (4) evaluate the contents of proanthocyanidins in all three knotweeds based on the total peak area; (5) identify proanthocyanidins in rhizomes of Bohemian and giant knotweeds using high performance thin-layer chromatography coupled to mass spectrometry (HPTLC–MS).

## 2. Results and Discussion

### 2.1. Qualitative Analyses of Flavan-3-ols and Proanthocyanidins with HPTLC

**Figure 1:**
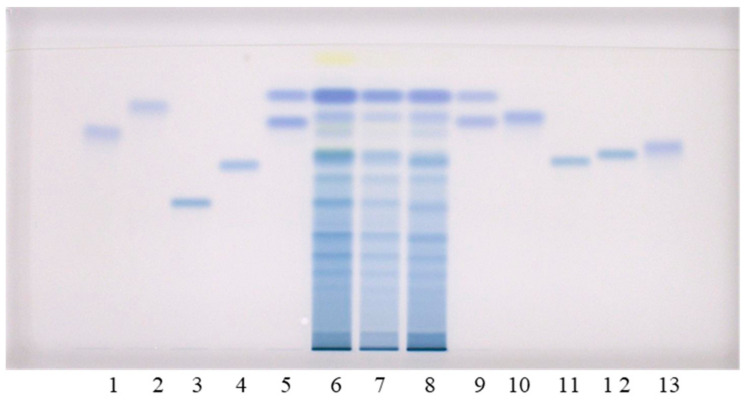
HPTLC chromatograms for the qualitative determination of flavan-3-ols and proanthocyanidins in STSs from rhizomes (2 μL, 50 mg/mL) of Japanese (track 6), Bohemian (track 7) and giant (track 8) knotweed based on standards. The HPTLC silica gel plate was developed with tol uene—acetone—formic acid (3:6:1, *v/v*) and documented at white light after derivatization with DMACA detection reagent. Applications of standards: (−)-gallocatechin gallate (0.2 µg; track 1), (−)-catechin gallate (0.2 µg; track 2), procyanidin C1 (0.3 µg; track 3), procyanidin B3 (0.2 µg; track 4), (−)-epicatechin (0.1 µg; track 5, higher R_F_), (−)-epigallocatechin (0.2 µg; track 5, lower R_F_), (+)-catechin (0.1 µg; track 9, higher R_F_) (−)-gallocatechin (0.2 µg; track 9, lower R_F_), (−)-epicatechin gallate (0.2 µg; track 10), procyanidin B1 (0.2 µg; track 11), procyanidin B2 (0.2 µg; track 12) (−)-epigallocatechin gallate (0.2 µg; track 13).

Qualitative analyses of flavan-3-ols and proanthocyanidins in the sample test solutions (STSs) prepared from rhizomes of Japanese, Bohemian and giant knotweed were performed using the high performance thin-layer chromatography (HPTLC) method on the HPTLC silica gel plates developed with toluene–acetone–formic acid (3:6:1, *v/v*) [9,16]. Post-chromatographic derivatization with 4-dimethylaminocinnamaldehyde (DMACA) reagent [17] enabled the detection of flavan-3-ols and proanthocyanidins, which was more selective than the detection before derivatization. We previously used this method in two studies: (1) an HPTLC/MS/MS study on flavan-3-ols and proanthocyanidins in rhizomes of Japanese knotweed [9]; and (2) an HPTLC study on flavan-3-ols and proanthocyanidins in leaves of Japanese, Bohemian and giant knotweed [18]. In this study, the following twelve standards were used for analyses: (+)-catechin, (−)-epicatechin, (−)-catechin gallate, (−)-epicatechin gallate, (−)-gallocatechin, (−)-epigallocatechin, (−)-gallocatechin gallate, (−)-epigallocatechin gallate, procyanidin B1, procyanidin B2, procyanidin B3 and procyanidin C1 (Figure 1). Eight of these twelve standards ((−)-catechin gallate (track 2), (−)-epicatechin gallate (track 10), (−)-gallocatechin gallate (track 1), (−)-epigallocatechin gallate (track 13), procyanidin B1 (track 11), procyanidin B2 (track 12), procyanidin B3 (track 4) and procyanidin C1 (track 3)) were separated from each other (Figure 1). The eight standards were also separated from the two pairs of unseparated standards (Figure 1). The first of the two pairs of unseparated standards includes (+)-catechin (track 9, higher R_F_) and (−)-epicatechin (track 5, higher R_F_). The second pair includes (−)-gallocatechin (track 9, lower R_F_) and (−)-epigallocatechin (track 5, lower R_F_). As shown in Figure 1, compounds (−)-epicatechin gallate (track 10), (−)-gallocatechin gallate (track 1), dimer procyanidin B2 (track 12) and trimer procyanidin C1 (track 3) were detected as intensive blue bands in STSs prepared from Japanese (track 6), Bohemian (track 7) and giant (track 8) knotweed rhizomes.

Additionally, intensive blue bands, present in all STSs from rhizomes (tracks 6–8), at the R_F_ of standards of monomers ((+)-catechin and (−)-epicatechin) confirmed the presence of monomers in rhizomes of all studied knotweeds (Figure 1). Procyanidin B1 (track 11) and procyanidin B3 (track 4) were only detected in STS from giant knotweed (Figure 1, tracks 6–8). Based on other standards (−)-catechin gallate (track 2), (−)-gallocatechin (track 9, lower R_F_), (−)-epigallocatechin (track 5, lower R_F_) and (−)-epigallocatechin gallate (track 13) were not detected in STSs of the three knotweed rhizomes (Figure 1, tracks 6–8).

**Figure 2:**
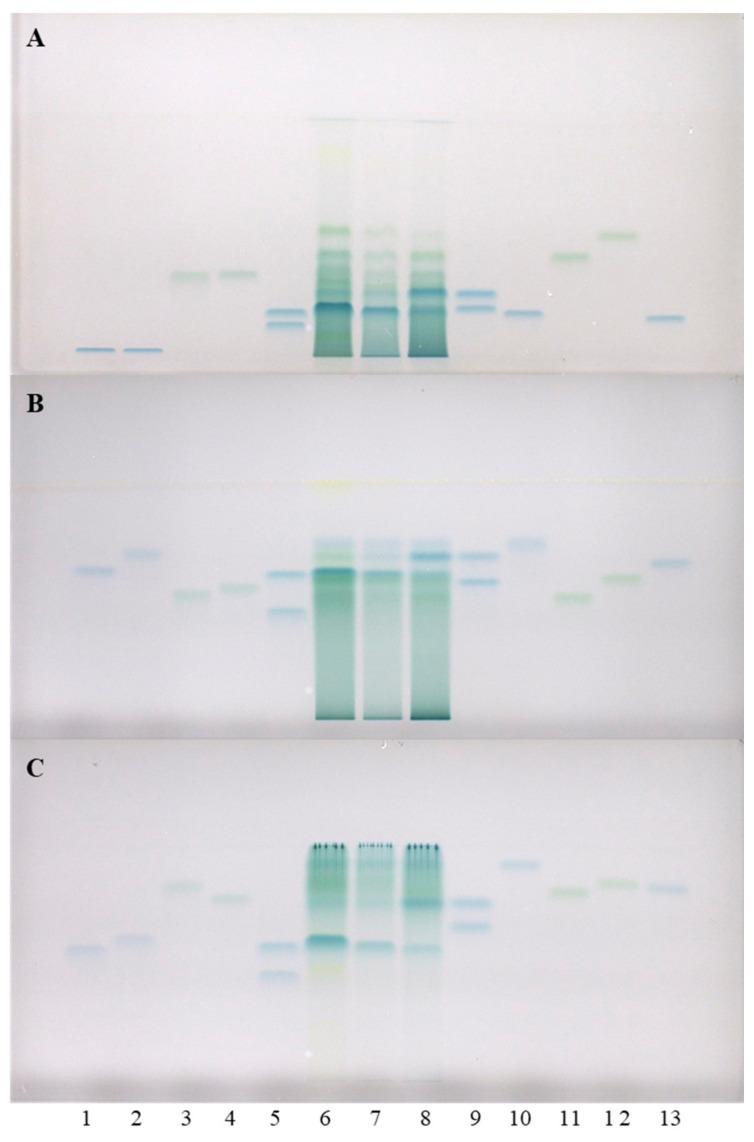
HPTLC chromatograms for the qualitative determination of flavan-3-ols and proanthocyanidins in STSs from rhizomes (1 μL, 50 mg/mL) of Japanese (track 6), Bohemian (track 7) and giant (track 8) knotweed based on standards. The HPTLC cellulose plates were developed with water (**A**), 1-propanol–water–acetic acid (4:2:1, *v/v*) (**B**), 1-propanol–water–acetic acid (20:80:1, *v/v*) (**C**), and documented at white light after derivatization with DMACA detection reagent. The ap plications of standards: (−)-gallocatechin gallate (60 ng; track 1), (−)-catechin gallate (60 ng; track 2), procyanidin C1 (150 ng; track 3), procyanidin B3 (100 ng; track 4), (−)-epicatechin (50 ng; track 5, higher R_F_), (−)-epigallocatechin (60 ng; track 5, lower R_F_), (+)-catechin (50 ng; track 9, higher R_F_), (−)-gallocatechin (60 ng; track 9, lower R_F_), (−)-epicatechin gallate (60 ng; track 10), procyanidin B1 (120 ng; track 11), procyanidin B2 (90 ng, track 12) (−)-epigallocatechin gallate (60 ng; track 13).

To further examine the obtained results, additional analyses of all three STSs from rhizomes were performed on HPTLC cellulose plates, using our previously developed methodology [17,19,20]. This methodology includes three developing solvents (water [17,19,20], 1-propanol–water–acetic acid (4:2:1, *v/v*) [17,20] and 1-propanol–water–acetic acid (20:80:1, *v/v*) [20,21,22]) and post-chromatographic derivatization with DMACA reagent [17]. This approach provided complementary results. The two pairs of standards (1st: (+)-catechin (track 9, higher R_F_) and (−)-epicatechin (track 5, higher R_F_); 2nd: (−)-gallocatechin (track 9, lower R_F_) and (−)-epigallocatechin (track 5, lower R_F_)) that were not separated on HPTLC silica gel plates, were separated on the HPTLC cellulose plate developed with water (Figure 2). Furthermore (+)-catechin and (−)-epicatechin were separated from all other standards on the HPTLC cellulose plates which were developed with water (Figure 2A) and 1-propanol–water–acetic acid (4:2:1 (*v/v*), Figure 2B). As shown in Figure 2, (+)-catechin (track 9, higher R_F_), (−)-epicatechin (track 5, higher R_F_), (−)-epicatechin gallate (track 10) and procyanidin B2 (track 12) were detected in Japanese (track 6), Bohemian (track 7) and giant (track 8) knotweed on the HPTLC cellulose plates developed with all three developing solvents. (−)-catechin gallate (track 2) was detected in STSs of all three knotweeds only on the plates developed with 1-propanol–water–acetic acid (4:2:1 (*v/v*), Figure 2B), and in STS of Japanese knotweed on the plate developed with 1-propanol–water–acetic acid (20:80:1 (*v/v*), Figure 2C). (−)-Gallocatechin (Figure 2A, track 9, lower R_F_) was detected in all three knotweeds only on the plates developed with water. Procyanidin B3 (track 4) was also detected in all three knotweeds only on the plate developed with water (Figure 2A), and was additionally detected in giant knotweed on the plate developed with 1-propanol–water–acetic acid (20:80:1 (*v/v*), Figure 2C). (−)-Epigallocatechin (Figure 2, track 5, lower R_F_) was not detected in any of the studied STSs from rhizomes. (−)-Gallocatechin gallate was detected in all three knotweeds, but only on the plates developed with the developing solvents containing 1-propanol–water–acetic acid (Figure 2B,C) and not on the plate developed with water (Figure 2A). (−)-Epigallocatechin gallate (track 13) was detected in all three knotweeds, but only on the plates developed with 1-propanol–water–acetic acid (20:80:1 (*v/v*), Figure 2C). Procyanidin B1 (track 11) was detected in all three knotweeds on the plates developed with water (Figure 2A) and 1-propanol–water–acetic acid (4:2:1 (*v/v*), Figure 2B), and only detected in giant knotweed on the plate developed with 1-propanol–water–acetic acid (20:80:1 (*v/v*), Figure 2C). Procyanidin C1 (track 3) was detected in all three STSs on the HPTLC cellulose plates developed with 1-propanol–water–acetic acid (4:2:1 (*v/v*), Figure 2B and 20:80:1 (*v/v*), Figure 2C). Procyanidin C1 was also detected in Bohemian and giant knotweed on the plate developed with water (Figure 2A).

Table 1 summarizes the results of the HPTLC analyses obtained with all four chromatographic systems involving two HPTLC stationary phases (silica gel and cellulose) in combination with the developing solvents used (toluene–acetone–formic acid (3:6:1, *v/v*) for silica gel plates (Figure 1); water and 1-propanol–water–acetic acid in different ratios (4:2:1, *v/v*; 20:80:1, *v/v*) for cellulose plates (Figure 2)). Data from Table 1 show that complementary methods are crucial to avoid reporting false positive results. In certain cases, as illustrated in Table 1, different combinations of the stationary phase and the developing solvent gave different results. The final confirmation of compounds in STSs was only given to those compounds which were detected using both stationary phases (column “Both” in Table 1) and all developing solvents. Based on these criteria we confirmed the presence of (+)-catechin, (−)-epicatechin, (−)-epicatechin gallate and procyanidin B2 (dimer) in rhizomes of Japanese, Bohemian and giant knotweed, and the presence of procyanidin B1 (dimer) and procyanidin C1 (trimer) in rhizomes of giant knotweed. (+)-Catechin and (−)-epicatechin were previously found in Japanese [8,15] and giant knotweed rhizomes [8]. In our recent study we isolated (+)-catechin, (−)-epicatechin, (−)-epicatechin gallate, procyanidin B1, procyanidin B2, procyanidin B3, proanthocyanidin B dimer gallate and some other compounds from rhizomes and bark of the rhizomes of Japanese knotweed [15].

To the best of our knowledge this is the first report on (+)-catechin, (−)-epicatechin, (−)-epicatechin gallate and procyanidin B2 (dimer) in Bohemian knotweed rhizomes. This is also the first report on (−)-epicatechin gallate, procyanidin B1 (dimer), procyanidin B2 (dimer) and procyanidin C1 (trimer) in giant knotweed rhizomes.

### 2.2. Chromatographic Fingerprinting (HPTLC) of Proanthocyanidins with Densitometry and Image Analysis

**Figure 3:**
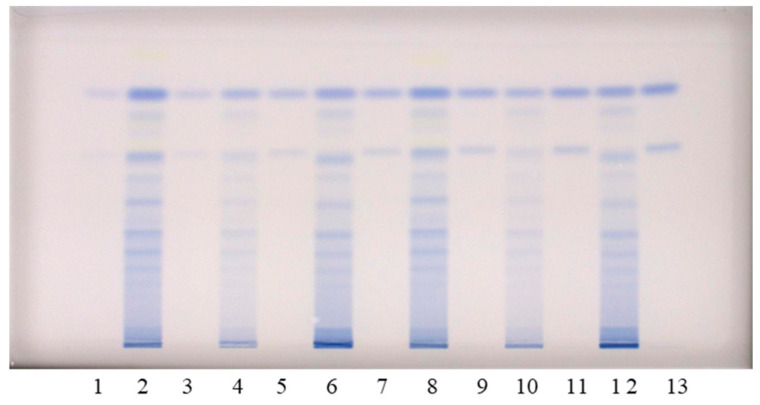
HPTLC chromatograms used for the quantitative determination of proanthocyanidins in STSs from rhizomes (1 μL, 50 mg/mL) of Japanese (tracks 2 and 8), Bohemian (tracks 4 and 10) and giant (tracks 6 and 12) knotweed and standard solutions of (−)-epicatechin and procyanidin B2. The HPTLC silica gel plate was developed with toluene–acetone–formic acid (3:6:1, *v/v*) and documented at white light after derivatization with DMACA detection reagent. The applications of (−)-epicatechin and procyanidin B2 standard solutions: track 1: 30 ng; track 3: 40 ng; track 5: 60 ng; track 7: 80 ng; track 9: 100 ng; track 11: 120 ng; track 13: 150 ng.

**Figure 4:**
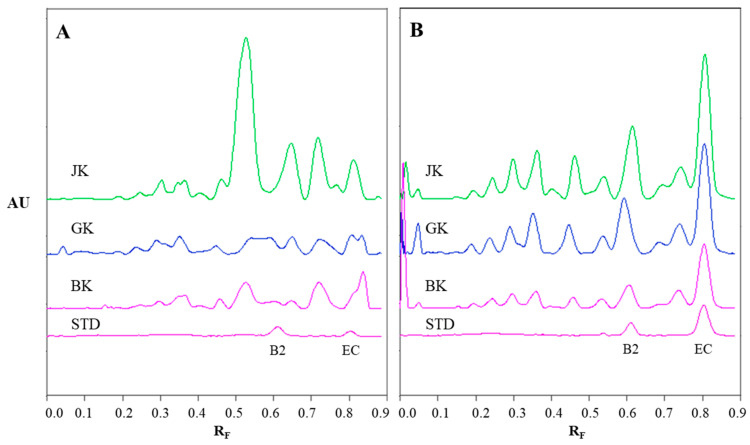
The densitograms of STSs (1 μL, 50 mg/mL) from rhizomes of Japanese (JK), giant (GK) and Bohemian (BK) knotweed and standard solutions (STD, 40 ng) of (−)-epicatechin (EC) and procyanidin B2 (B2) scanned in absorption/reflectance mode at 280 nm before the derivatization (**A**) and at 655 nm after the derivatization with DMACA reagent (**B**). The HPTLC silica gel plate was developed with toluene–acetone–formic acid (3:6:1, *v/v*).

The chromatographic fingerprinting of proanthocyanidins in STSs from rhizomes of Japanese, Bohemian and giant knotweed on HPTLC silica gel plate was performed with densitometry and image analysis. Only densitometry was applied for the chromatographic fingerprinting of proanthocyanidins on the developed plates (before post-chromatographic derivatization). Additionally, densitometry and image analysis were used for fingerprinting after post-chromatographic derivatization with DMACA reagent (Figure 3). To detect underivatized proanthocyanidins and their derivatives, formed when proanthocyanidins react with DMACA reagent, densitometric scanning was performed in the absorption/reflectance mode at two wavelengths 280 nm (Figure 4A) and 655 nm (Figure 4B), re spectively. As evident from the densitograms of (−)-epicatechin and procyanidin B2 standards before (Figure 4A) and after (Figure 4B) post-chromatographic derivatization, derivatization with DMACA reagent significantly enhanced the sensitivity of the HPTLC method. This enhancement of the sensitivity is shown in a considerable increase in the heights of the peaks in the densitograms scanned after the derivatization for both standards and all STSs at R_F_ values for monomers (R_F_ of (−)-epicatechin) and dimers (R_F_ of procyanidin B2) of B-type proanthocyanidins (Figure 4B). The densitograms of all STSs contain two peaks at the same R_F_ values as the standard solutions of (−)-epicatechin and procyanidin B2. These peaks confirm that Japanese, Bohemian and giant knotweed contain monomers and dimers of B-type proanthocyanidins (Figure 4B). Comparisons of the densitograms of each of the three STSs from rhizomes recorded on the same plate before (Figure 4A) and after (Figure 4B) derivatization show differences in the chromatographic fingerprints. These differences can be explained by the fact that at 280 nm the light was also absorbed by other compounds (e.g., phenolic acids, other flavonoids) which are present together with flavan-3-ols and proanthocyanidins in all three knotweed STSs. Therefore, peaks at other R_F_ values are also visible.

**Figure 5:**
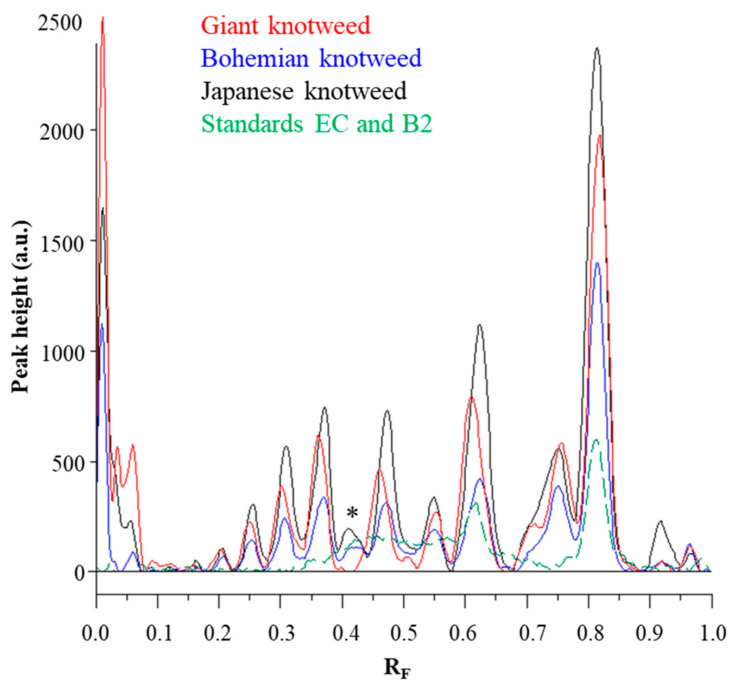
Comparisons of the videodensitogram of standards (−)-epicatechin (EC; R_F_ = 0.82) and procyanidin B2 (B2; R_F_ = 0.63) (30 ng; dashed green line) with the videodensitograms of the fingerprint profiles of STSs (1 μL, 50 mg/mL) from rhizomes of Japanese (black line), Bohemian (blue line) and giant knotweed (red line). The videodensitograms were obtained in absorption mode by image analysis of the HPTLC silica gel plate after the development with toluene–acetone–formic acid (3:6:1, *v/v*) and after the derivatization with DMACA detection reagent. The asterisk (*) indicates the peaks that are specific to Japanese and Bohemian knotweed rhizomes.

**Figure 6:**
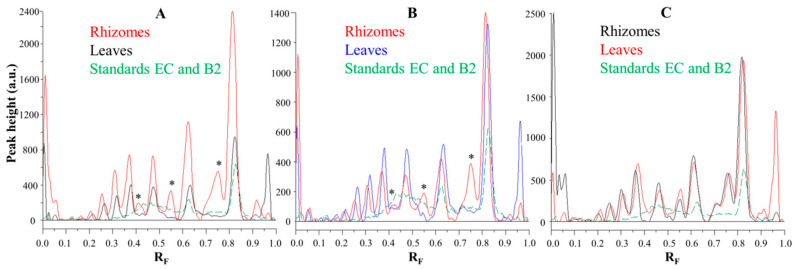
Comparisons of the videodensitogram fingerprint profiles of STSs (1 μL, 50 mg/mL) from leaves and rhizomes of the same knotweed species (Japanese (**A**), Bohemian (**B**) and giant (**C**) knotweed) with the videodensitogram of standards (−)-epicatechin (EC; R_F_ = 0.82) and procyanidin B2 (B2; R_F_ = 0.63) (30 ng; dashed green line). The videodensitograms were obtained in absorption mode by image analysis of the HPTLC silica gel plates after the development with toluene–acetone–formic acid (3:6:1, *v/v*) and after the derivatization with DMACA detection reagent. The asterisks (*) in dicate peaks that are only present in the rhizomes of Japanese and Bohemian knotweed.

Chromatographic fingerprinting of proanthocyanidins with image analysis was performed at white light after post-chromatographic derivatization with DMACA reagent. Image analysis of the underivatized proanthocyanidins could not be performed at 280 nm due to the technical limitations of the DigiStore 2 documentation system, which in the UV zone only permits the use of wavelengths of 366 and 254 nm. Therefore, the chromatographic fingerprinting of proanthocyanidins with image analysis was performed only after post-chromatographic derivatization with DMACA reagent. After post-chromatographic derivatization the images of the HPTLC silica gel plates were captured at white light illumination. The images (Figure 3) were then converted to videodensitograms (Figure 5 and Figure 6) in absorption mode. The videodensitograms of STSs from rhizomes of all three knotweeds show peaks at R_F_ values of (−)-epicatechin and procyanidin B2 standards, which confirms the presence of monomers and dimers in all knotweed STSs (Figure 5). Comparisons of the videodensitograms of STSs from rhizomes showed that Japanese and Bohemian knotweed have equal qualitative profiles, which also included the peak at R_F_ 0.41, which was not detected in giant knotweed (Figure 5). Additionally, from the peak heights in vid eodensitograms of all STSs it is evident that Japanese knotweed is the richest source of proanthocyanidins (Figure 5). The chromatographic fingerprints of rhizomes of Japanese, Bohemian and giant knotweed were also compared with the chromatographic fingerprints of leaves obtained from the respective plant species and collected at the same locations [18] as the rhizomes. The STSs of leaves were prepared as described in our previous study [18].

Comparisons of the chromatographic fingerprints of the rhizomes and leaves of Japanese (Figure 6A), Bohemian (Figure 6B) and giant (Figure 6C) showed that within the individual knotweed species giant knotweed rhizomes and leaves have the most similar fingerprints (Figure 6C), while the fingerprints of Japanese (Figure 6A) and Bohemian (Figure 6B) knotweed rhizomes and leaves differ in the peaks at R_F_ 0.41, 0.55, and 0.75 which are only present in rhizomes (Figure 6A,B). The smallest differences in the heights of peaks at R_F_ values of (−)-epicatechin and procyanidin B2 standards were observed between the videodensitograms of rhizomes and leaves of giant knotweed (Figure 6C). The biggest differences at these R_F_s were observed in the heights of peaks between rhizomes and leaves of Japanese knotweed (Figure 6A), with significantly higher peaks for rhizomes. In the case of Bohemian knotweed the differences between the peak heights of rhizomes and leaves are less pronounced. However, for rhizomes the peak at R_F_ of (−)-epicatechin is higher than for leaves, and the peak at R_F_ of procyanidin B2 standard is lower than for leaves.

### 2.3. Quantification of Proanthocyanidins

HPTLC combined with image analysis was applied for the quantitative determination of proanthocyanidins with the focus on: (1) differences in the contents of monomers and dimers in rhizomes of all three knotweeds; (2) total proanthocyanidins content. Im ages of the chromatograms were converted to videodensitograms, which were integrated using VideoScan software. Limits of detection (LOD), limits of quantification (LOQ) and the stability of the derivatized standards of (−)-epicatechin (100 ng) and procyanidin B2 (100 ng) on the HPTLC silica gel plates were described in our previous study [18]. STSs from rhizomes were analyzed on an HPTLC silica gel plate (Figure 4) using equal amounts (30, 40, 60, 80, 100, 120 and 150 ng per plate) of (−)-epicatechin and procyanidin B2 standards for the calibration curves. Equal volumes of each STS were applied twice on the plate (once on the left and once on the right side of the plate). For quantification the mean value of the peak areas from both applications (at the same R_F_) was cal culated (data-pair technique), which reduced the effect of inequity of the stationary phase. The equations of the calibration curves based on peak areas of standards (−)-epicatechin and procyanidin B2 were used to calculate the contents of monomers (flavan-3-ols) and procyanidin dimers, respectively, in rhizomes of Japanese, Bohemian and giant knotweed (Table 2). This is the first report about the quantification of the total content of monomers and dimers of proanthocyanidins in rhizomes of Japanese, Bohemian and giant knotweed. Other authors reported only the contents of separate monomers ((+)-catechin and (−)-epicatechin) [7,8,23] and B-type dimers (specified as “procyanidin dimer B”) [7,8] in the rhizomes of Japanese [7,8,23] and giant [7,8] knotweed. The total contents of monomers and dimers expressed per dry mass of the plant material (DM) for all STSs were the highest in Japanese knotweed rhizomes (5.80 kg/t DM) and the lowest in Bohemian knotweed rhizomes (2.61 kg/t DM), while it was 4.53 kg/t DM in gi ant knotweed rhizomes (Table 2). It was determined that the content of monomers (2.36 kg/t DM) in giant knotweed rhizomes was equal to that reported for its leaves in our previous study [18]. The contents of dimers in rhizomes (2.17 kg/t DM) and leaves (2.06 kg/t DM) [18] of giant knotweed were also comparable. The content of monomers ob tained in rhizomes (1.52 kg/t DM) was slightly higher than in leaves (1.39 kg/t DM) [18] of Bohemian knotweed. Even more pronounced differences were observed between the contents of dimers in rhizomes (1.09 kg/t DM) and leaves (1.40 kg/t DM) [18] of Bohemian knotweed. The differences between Japanese knotweed rhizomes and leaves were significant. Rhizomes are more than three times richer in monomers (2.99 kg/t DM) than leaves (0.84 kg/t DM) [18] and almost three times richer in dimers (2.81 kg/t DM) than leaves (0.99 kg/t DM) [18].

Two other groups of authors reported separate contents of two monomers ((+)-catechin, (−)-epicatechin) [7,8,23]) and B-type dimers (specified as “procyanidin di mer B” [7,8]) per 100 g DM. Using their data we calculated contents of monomers and dimers in their samples of rhizomes [7,8,23] and leaves [7,8] of Japanese [7,8,23] and giant [7,8] knotweed. The first of these two groups reported 118 to 215 mg catechin/100 g DM and from 212 to 265 mg of epicatechin/100 g DM of the samples of Japanese knotweed rhizomes [23]. Since both compounds are monomers, we calculated that they determined from 330 to 480 mg of monomers/100 g DM of Japanese knotweed rhizomes, which is slightly higher than was determined in our study (Table 2).

The second group of authors determined 82 mg of monomers (8 mg (+)-catechin, 74 mg (−)-epicatechin) per 100 g DM of rhizomes, and 475 mg of monomers (145 mg (+)-catechin, 330 mg (−)-epicatechin) per 100 g DM of leaves of Japanese knotweed [8]. The monomers content in rhizomes was 3.6 times lower than in our study (Table 2), while the monomers content in leaves was 5.6 times higher than in our previous study on Japanese knotweed leaves [18]. In giant knotweed they found 190 mg of monomers (21 mg (+)-catechin, 169 mg (−)-epicatechin) per 100 g DM of rhizomes and 449 mg of monomers (111 mg (+)-catechin, 338 mg (−)-epicatechin) per 100 g DM of leaves [8]. The monomers content in giant knotweed rhizomes was 1.2 times lower than in our study (Table 2), while the monomers content in leaves was 1.9 times higher than in our study on giant knotweed leaves [18]. Separate contents of B-type dimers (specified as “procyanidin dimer B”) were also determined in rhizomes and leaves of Japanese and giant knotweed [8]. Using the contents of the two dimers in rhizomes and the three dimers in leaves of both species we calculated the following dimers contents in 100 g of DM: 184 mg in rhi zomes and 1251 mg in leaves of Japanese knotweed; 206 mg in rhizomes and 1125 mg in leaves of giant knotweed [8]. The dimers content in Japanese knotweed rhizomes was 1.5 times lower than in our study (Table 2), while the dimers content in leaves was 12.6 times higher than in our study on Japanese knotweed leaves [18]. The dimers content in giant knotweed rhizomes was comparable with that obtained in our study (Table 2), while the dimers content in leaves was 5.5 times higher than in our previous study on leaves [18].

The second group of authors performed an additional study on Japanese and giant knotweed [7], in which they used the same analytical method as in [8], and reported separate contents of (+)-catechin, (−)-epicatechin and three B-type dimers (specified as “procyanidin dimer B”) in rhizomes and leaves of both species. The contents in 100 g of dry Japanese knotweed materials were as follows: 360 mg of monomers (90 mg (+)-catechin, 270 mg (−)-epicatechin) and 970 mg of dimers in rhizomes, and 320 mg of monomers (190 mg (+)-catechin, 130 mg (−)-epicatechin) and 520 mg of dimers in leaves [7]. The contents in 100 g of dry giant knotweed materials were: 840 mg of monomers (220 mg (+)-catechin, 620 mg (−)-epicatechin) and 730 mg of dimers in rhizomes, and 270 mg of monomers (140 mg (+)-catechin, 130 mg (−)-epicatechin) and 450 mg of dimers in leaves of giant knotweed [7]. The content of monomers in Japanese knotweed rhizomes was 4.4 times higher than in [8] and 1.2 times higher than in our study (Table 2), while the monomers content in leaves was 1.5 times lower than in [8] and 3.8 times higher than in our study on leaves [18]. The dimers content in Japanese knotweed rhi zomes was 5.3 times higher than in [8] and 3.5 times higher than in our study (Table 2), while the monomers content in leaves was 2.4 times lower than in [8] and 5.3 times higher than in our study on leaves [18]. The monomers content in giant knot weed rhizomes was 4.4 times higher than in [8] and 3.6 times higher than in our study (Table 2), while the monomers content in leaves was comparable to that obtained in our study on leaves [18] and 1.7 times lower than in [8]. The dimers content in giant knotweed rhizomes was 3.5 times higher than in [8] and 3.4 times higher than in our study (Table 2), while the dimers content in leaves was 2.5 times lower than in [8] and 2.2 times higher than in our study on leaves [18]. The differences in the contents obtained by the various studies arise from numerous factors, which influence the biosynthesis of these natural compounds. These factors include: the phenological stage of the plant, the position on the plant (for example leaves), the abiotic and biotic factors such as the growth conditions (temperature, soil nutrition supply, water supply, light intensity), infections with pests or diseases, attacks by herbivores and others.

**Figure 7:**
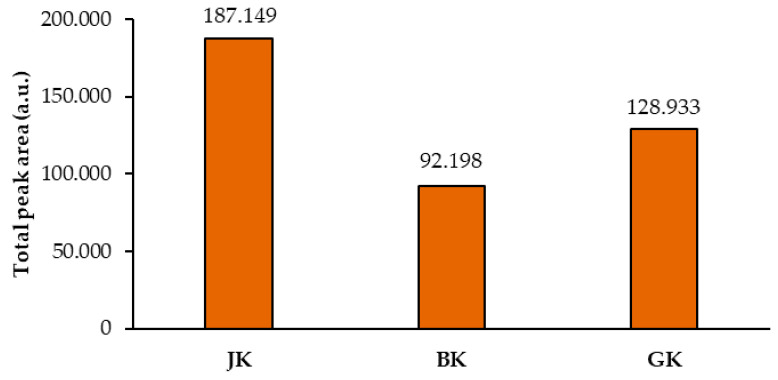
Comparison of the means of the total peak areas of proanthocyanidins (blue bands in chromatograms) for STSs from rhizomes of Japanese (JK), Bohemian (BK), and giant (GK) knotweed. The mean of the total peak areas was calculated from the total peak areas of the videodensitograms of two equal applications of the same STS on the HPTLC silica gel plate (Figure 3) after the development with toluene–acetone–formic acid (3:6:1, *v/v*) and after the derivatization with DMACA detection reagent.

Total peak areas of the videodensitograms of the two equal applications of the same STS on HPTLC silica gel plates were used to calculate the mean of the total peak areas of proanthocyanidins (all blue bands in chromatograms). This provided additional information about proanthocyanidins in all STSs (Figure 7). The comparison of the mean values of the total peak areas of proanthocyanidins proved the highest contents of proanthocyanidins in Japanese knotweed and the lowest in Bohemian knotweed rhizomes (Figure 7), with the difference between both for the factor 2, and the difference between Japanese and giant knotweed for the factor 1.5. Similar differences were also obtained in our previous study of leaves of all three species, where the highest content determined in giant knotweed leaves was 1.8 and 2.2 times higher than in Bohemian and Japanese knotweed, respectively [18].

### 2.4. HPTLC–MS/MS Characterization of Flavan-3-ols and Proanthocyanidins

HPTLC–MS/MS analyses of flavan-3-ols and proanthocyanidins in STSs from Bohemian and giant knotweed rhizomes were performed using the same method as in our previous studies of flavan-3-ols and proanthocyanidins in Japanese knotweed rhizomes [10] and Japanese, Bohemian and giant knotweed leaves [18]. Acetonitrile was used for the pre-development and development of the HPTLC diol F_254S_ plates. The left incised (but not cut) edge of each developed plate was broken off and derivatized with DMACA reagent, which was done to detect proanthocyanidins bands (not visible on the developed plate) and to prevent their possible degradation (due to heating) before de tection with MS. This derivatized part of the plate was then put next to the underivatized part of the plate. The blue-colored proanthocyanidins zones were then used for the proper positioning of the elution head of the TLC–MS interface on the parallel zones with the same R_F_ on the underivatized part of the plate. Only the underivatized zones were eluted from the plate and transferred to the MS detector for acquisition of the MS, MS^2^ and MS^3^ spectra.

**Figure 8:**
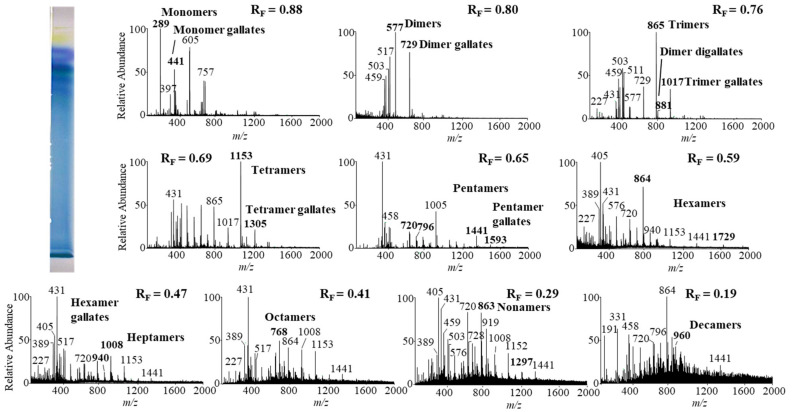
The MS spectra obtained by HPTLC—MS analysis of the STS from Bohemian knotweed rhizomes on HPTLC diol F_254S_ plate pre-developed and developed with acetonitrile. The bolded *m/z* values in the MS spectra belong to B-type proanthocyanidins and their gallates eluted from the underivatized part of the plate with acetonitrile–methanol (2:1, *v/v*). A narrow derivatized (DMACA reagent) part of the plate with blue-colored proanthocyanidins zones was used for the proper positioning of the elution head of the TLC—MS interface.

**Figure 9:**
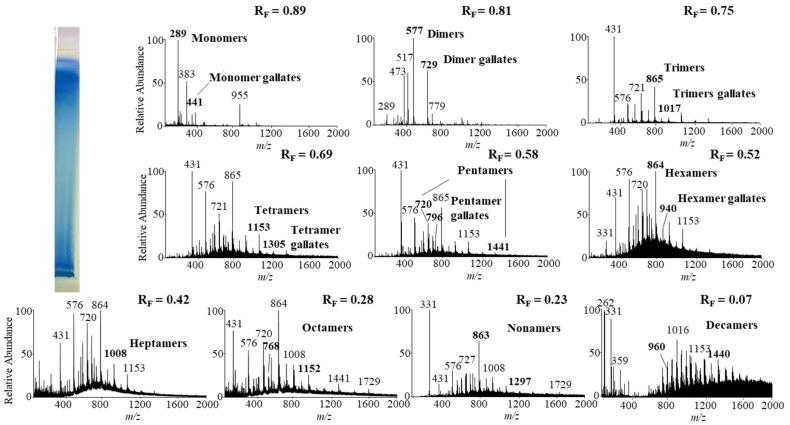
The MS spectra obtained by HPTLC–MS analysis of STS from giant knotweed rhizomes on the HPTLC diol F_254S_ plate pre-developed and developed with acetonitrile. The bolded *m/z* values in the MS spectra belong to B-type proan thocyanidins and their gallates eluted from the underivatized part of the plate with acetonitrile–methanol (2:1, *v/v*). A narrow derivatized (DMACA reagent) part of the plate with blue-colored proanthocyanidins zones was used for the proper positioning of the elution head of the TLC–MS interface.

Monomers, monomer gallates, dimers and trimers were identified by comparing the fragmentation patterns with those of their respective commercial standards (−)-epicatechin, (−)-epicatechin gallate, procyanidin B2 and procyanidin C1. Standards of oligomers with higher degrees of polymerization (tetramers up to decamers), their gal lates and digallates are not commercially available. Therefore, tetramers to decamers, as well as their gallates and digallates, were tentatively identified based on their MS spectra (Figure 8 and Figure 9, Table 3) and comparisons of the obtained and published frag mentation patterns. Pentamers to decamers and their gallates were identified as double- or triple-charged deprotonated molecular ions due to the limitations of the mass range of the MS detector.

Table 3 compares data from this study with literature data about proanthocyanidins identified in rhizomes [7,8,9,10,12,13,14] and leaves [7,8,18] of Japanese [7,8,9,10,12,13,14,18], Bohemian [12,14,18] and giant [7,8,12,13,14,18] knotweed by using HPTLC–MS/MS [9,10,18] and HPLC–MS methods [7,8,12,13,14]. In this study we identified all B-type pro anthocyanidins from monomers to decamers (monomers, dimers, trimers, tetramers, pentamers, hexamers, heptamers, octamers, nonamers and decamers), as well as some of their gal lates (monomer gallates, dimer gallates, dimer digallates, trimer gallates, te tramer gallates, pentamer gallates and hexamer gallates) in rhizomes of Bohemian (Figure 8, Table 3) and giant knotweed (Figure 9, Table 3). To the best of our knowledge we were the first to identify pentamer gallates, hexamers, hexamer gallates, nonamers and decamers in rhizomes of Bohemian and giant knotweed. All these proanthocyanidins and their gallates were previously reported in our studies of Japanese knotweed rhi zomes by using the same HPTLC–MS/MS method [10] and by HPTLC–MS/MS method on HPTLC silica gel plates [9]. Rhizomes of all three knotweeds have the same chemical profile of proanthocyanidins with respect to the degree of polymerization and also with respect to gallates (Table 3). Rhizomes of all three knotweeds also have the same chemical profile of proanthocyanidins with respect to the degree of polymerization as was discovered for leaves of Japanese, Bohemian and giant knotweed in our previous study [18] (Table 3). Additionally, rhizomes of all three knotweeds and leaves of giant knotweed have the same chemical profile of proanthocyanidins with respect to gallates (Table 3). Only a few gallates are present in the profiles of leaves of Japanese knotweed (monomer gallates and dimer gallates) and Bohemian knotweed (monomer gallates, di mer gallates and dimer digallates) (Table 3). Within the individual species of the stud ied knotweeds, only giant knotweed rhizomes and leaves have the same chemical profile of proanthocyanidins with respect to the degree of polymerization and also with respect to gallates (Table 3).

The investigations on proanthocyanidins in knotweeds were performed on Japanese knotweed rhizomes [7,8,9,10,12,13,14] using HPLC–MS [7,8,12,13,14] or HPTLC–MS [9,10] methods (Table 3). Literature data about proanthocyanidins identified (by using mass spectrometry) in Bohemian knotweed rhizomes are rather scarce (Table 3). There are only two publications. One of the publications provides data only about monomers and monomer gallate [12], the other in addition to monomers also reports dimers, dimer gal lates, dimer digallates, trimers, trimer gallates, trimer digallates, tetramer, tetramer gallates, pentamers, heptamers and octamers [14]. Both publications [12,14] report the same proanthocyanidins and their gallates in giant knotweed. Additional publications reported monomers [7,8,13] and monomer gallate [7,8,13], dimers [7,8], dimer gallates [13], dimer digallates [13] in giant knotweed (Table 3).

## 3. Materials and Methods

### 3.1. Chemicals

All chemicals were at least of analytical grade. Acetic acid, formic acid, hydrochloric acid (37%), toluene, 1-propanol, and 4-dimethylaminocinnamaldehyde (DMACA) were from Merck (Darmstadt, Germany). Acetone, ethanol as well as HPLC grade acetonitrile and methanol were purchased from Sigma-Aldrich (St. Louis, MO, USA). LC–MS grade acetonitrile and methanol used for MS analyses were from Fluka (Buchs, Switzerland). MilliQ 18.2 MΩ water (Millipore, Bedford, MA, USA) was also used.

Standard of (−)-epicatechin was obtained from Sigma-Aldrich and (+)-catechin from Carl Roth (Karlsruhe, Germany). Standards of procyanidin B1, procyanidin B2, procyanidin B3, procyanidin C1, (−)-catechin gallate, (−)-gallocatechin, (−)-gallocatechin gallate, (−)-epicatechin gallate, (−)-epigallocatechin and (−)-epigallocatechin gallate were obtained from Extrasynthèse (Genay, France).

### 3.2. Preparation of Standard Solutions

Stock solutions of standards (0.1 mg/mL) were individually prepared in methanol. Separate working solutions (10 μg/mL) were prepared by diluting of stock solutions with methanol. All standard solutions were stored in amber glass storage vials at −80 °C.

### 3.3. Plant Materials

Rhizomes (a subterranean stem) of Japanese knotweed (*Fallopia japonica* Houtt.), Bohemian knotweed (*Fallopia* × *bohemica* (Chrtek & Chrtkova) J.P. Bailey) and giant knot weed (*Fallopia sachalinensis* F. Schmidt) were collected at the beginning of September 2018 in Ljubljana (Slovenia).

Rhizomes were first washed with tap water to remove soil, dried on air, cut into smaller parts, frozen with liquid nitrogen and lyophilized (Micro Modulyo, IMAEdwards, Bologna, Italy) for 48 h at −50 °C. Lyophilized materials were again individually frozen with liquid nitrogen before they were crushed and pulverized with Mikro-Dismembrator S (Sartorius, Göttingen, Germany) at a frequency of 1700 min^−1^ for 1 min. Lyophilized powdered materials were stored at −20 °C before being used for the preparation of sample test solutions (STSs).

### 3.4. Preparation of Sample Test Solutions (STSs) from Rhizomes

STSs (50 mg/mL) from rhizomes were prepared separately. Powdered lyophilized plant material was dispersed in 4 mL of 70% aqueous acetone. The resulting suspension was vortexed (1 min at 2800 rpm; IKA lab dancer, Sigma-Aldrich) and then centrifuged at 4500 rpm (Centric 322 A, Tehtnica, Železniki, Slovenia) for 5 min. The supernatant was fil tered through a 0.45 µm polyvinylidene fluoride (PVDF) membrane filter (Millipore, Billerica, MA, USA) into amber glass storage vials, which were stored at −20 °C. After thawing, smaller particles were present on the bottom of some STSs’ vials. The solutions were then centrifuged for 3 min at 13,400 rpm (MiniSpin centrifuge, MiniSpin, Eppendorf, Hamburg, Germany) and the supernatants (STSs from rhizomes of all three knotweeds) were transferred into GC vials. These undiluted STSs were then used for HPTLC and HPTLC–MS/MS analyses.

### 3.5. HPTLC with Image Analysis and Densitometry

HPTLC analyses were performed on un-predeveloped 20 cm × 10 cm glass backed HPTLC silica gel plates (Merck, Art. No. 1.05641) and HPTLC cellulose plates (Merck, Art. No. 1.05786). An automatic TLC Sampler 4 (Camag, Muttenz, Switzerland) was used to apply STSs and all standard solutions on the plates. Applications were performed as 10 mm bands, 8 mm from the bottom of the plate and 25 mm from the left edge.

For the qualitative analysis on the HPTLC silica gel plate and HPTLC cellulose plates we applied different volumes of STSs (50 mg/mL) and solutions of standards (0.01 mg/mL; (−)-epicatechin, (+)-catechin, (−)-epicatechin gallate, (−)-epigallocatechin gallate, (−)-epigallocatechin, (−)-catechin gallate, (−)-gallocatechin, (−)-gallocatechin gallate, procyanidin B1, procyanidin B2, procyanidin B3 and procyanidin C1).

For chromatographic fingerprinting and quantitative analyses on the HPTLC silica gel plate we applied lower volumes of STSs (1 μL; 50 mg/mL) and solutions of (−)-epicatechin and procyanidin B2 standards (3, 4, 6, 8, 10, 12 and 15 µL; 0.01 mg/mL). Each of the STSs from rhizomes was applied on the plate twice using a data-pair technique (one application on the left and the other on the right half of the plate).

The HPTLC silica gel plates were developed up to 9 cm using 10 mL of the developing solvent toluene–acetone–formic acid (3:6:1, *v/v*) [9], which was added only in one trough of an unsaturated twin-trough chamber (Camag) for 20 cm × 10 cm plates. The developing time was 25 min. The HPTLC cellulose plates were developed in a horizontal developing chamber (sandwich configuration; Camag) for 20 cm × 10 cm plates using the following developing solvents: water [17,19,20], 1-propanol–water–acetic acid (4:2:1, *v/v*) [17,20] and 1-propanol–water–acetic acid (20:80:1, *v/v*) [20,21,22].

After the development and drying in a stream of warm air for 1 min, post-chromatographic derivatization was performed by dipping the plates for 1 s into DMACA dipping detection reagent. DMACA reagent was prepared by dissolving 60 mg of DMACA in 13 mL of concentrated hydrochloric acid, which was made up to 200 mL with ethanol [17]. Dipping was followed by drying for 2 min in a stream of warm air.

Documentation of the images of the plates with a DigiStore 2 documentation system (Camag) at 366 nm and white light illumination was performed immediately after de velopment and 10 min after post-chromatographic derivatization. For the purpose of image analyses, the obtained images of the HPTLC plates captured at white light illumination after post-chromatographic derivatization were converted to a different format using WinCATS software and then converted to videodensitograms in absorption mode using VideoScan TLC/HPTLC Evaluation Software (Version 1.02.00) (Camag). Densitometric analyses were performed by a slit-scanning densitometer TLC Scanner 3 (Camag). Densitometric scanning was performed in the absorption/reflectance mode at 280 nm (before derivatization) or 655 nm (10 min after derivatization). The dimensions of the slit were: length 6 mm, width 0.3 mm; and the scanning speed 20 mm/s. All instruments were controlled by the winCATS software (Version 1.4.9.2001).

### 3.6. HPTLC–MS/MS Analyses

HPTLC–MS/MS analyses of Bohemian and giant knotweed were performed separate on HPTLC glass backed diol F_254S_ plates (20 cm × 10 cm, Merck, Art. No. 1.05636). The plates were predeveloped with acetonitrile [10], dried for 5 min in a stream of warm air and cut in 10 cm × 10 cm parts. Each plate was incised (but not cut) 15 mm from the left edge before application. STSs (Bohemian knotweed—125 μL giant knotweed—100 μL; both 50 mg/mL) were applied by Linomat 5 (Camag) as 60 mm bands, 8 mm from the bottom of the plate.

Plates were developed up to 9 cm with acetonitrile [10] in an unsaturated twin trough chamber (Camag) for 10 cm × 10 cm plates in 15 min. After development and drying for 1 min in a stream of warm air, the leftmost 15 mm part was broken off from the plate and only this part was dipped for 1 s into DMACA reagent for proanthocyanidin visualization (blue zones). Ten minutes after drying in a stream of warm air the derivatized part of the plate was attached to the underivatized part of the plate with scotch tape and documented by DigiStore 2 Documentation system. The blue zones were used for the proper positioning of the elution head of a TLC–MS interface (Camag) on the parallel zones with same R_F_ in the remaining part of the track on the underivatized part of the plate. Only the zones on the underivatized part of the plate were eluted from the plate and transferred into the MS detector for acquisition of first-order mass (MS), product ion (MS^2^) and (MS^3^) spectra.

A quaternary pump Accela Pump (part of the UHPLC system, Thermo Fisher Scientific, Waltham, MA, USA) and the TLC–MS interface with an oval elution head (4 mm × 2 mm) were used on-line to elute analytes from the plates into a LTQ Velos mass spectrometer with dual-pressure linear ion trap mass analyzer (Thermo Fisher Scientific). Acetonitrile–methanol (2:1, *v/v*) [9,10,24] at a flow rate of 0.2 mL/min was used as an eluent. An in-line filter (Idex, Health & Science, Oak Harbor, WA, USA) was installed between the TLC–MS interface and the ion source. For ionization of compounds a heated electrospray ionization (HESI) ion source was used in negative ion mode. The MS capillary temperature and heater temperature were 350 °C and 250 °C, respectively. Other MS conditions were as follows: sheath gas 35 a.u. (arbitrary units), auxiliary gas 10 a.u., sweep gas 0 a.u., spray voltage 3.5 kV, S-lens RF level 65 % and capillary voltage −38.8 V. MS spectra were acquired for 1 min in the *m/z* range 150–2000. The fragmentation of the parent ion was performed at 30% collision energy and the isolation width of 1.0 *m/z*. The data collected were evaluated with Xcalibur software (version 2.1.0). After the completion of the HPTLC–MS/MS analyses, the plate was documented with the DigiStore 2 system at white light illumination in order to connect the MS, MS^2^, and MS^3^ spectra with R_F_s on the plate.

## 4. Conclusions

This is the first report about: (1) comparison of HPTLC chromatographic fingerprints of rhizomes of Japanese, Bohemian and giant knotweed; (2) comparisons of chromatographic fingerprints for rhizomes and leaves of the same knotweed species; (3) quantifications of the total content of monomers (flavan-3-ols) and procyanidin dimers in rhi zomes of all three knotweed species; (4) evaluations of rhizomes of the three knotweed species based on comparisons of B-type proanthocyanidins total peak areas; (5) evaluations of the contents of proanthocyanidins in rhizomes of all three knotweeds based on the total peak area; (6) analyses and identification of all B-type proanthocyanidins from monomers to decamers and some of their gallates in Bohemian and giant knotweed using HPTLC–MS.

Monomers (+)-catechin, (−)-epicatechin, (−)-epicatechin gallate and dimer procyanidin B2 (dimer) were found in rhizomes of all three species. To the best of our knowledge this is the first report on detection of monomers (+)-catechin, (−)-epicatechin, (−)-epicatechin gallate and dimer procyanidin B2 in Bohemian knotweed rhizomes, and (−)-epicatechin gallate, dimers procyanidin B1 and procyanidin B2, as well as trimer procyanidin C1 in giant knotweed rhizomes.

This is the first report of identification of all B-type proanthocyanidins from monomers to decamers (monomers—flavan-3-ols, dimers, trimers, tetramers, pentamers, hexamers, heptamers, octamers, nonamers and decamers) and some of their gallates (monomer gallates, dimer gallates, dimer digallates, trimer gallates, tetramer gallates, pentamer gallates and hexamer gallates) in crude extracts from Bohemian knotweed and giant knotweed rhizomes. From these compounds above, pentamer gallates, hexamers, hexamer gallates, nonamers and decamers were for the first time identified in rhizomes of Bohemian and giant knotweed. We previously found all these proanthocyanidins also in Japanese knotweed rhizomes. Based on these findings, it can be concluded that Japanese, Bohemian and giant knotweed rhizomes have the same chemical profiles of proanthocyanidins with respect to the degree of polymerization and with respect to gallates. Japanese and Bohemian knotweed rhizomes have equal chromatographic fingerprint profiles, differing by one additional peak from the fingerprint profile of giant knotweed.

Within the individual species the fingerprints of rhizomes and leaves of giant knotweed have been found to be the most similar. The fingerprints of Japanese and Bohemian knotweed rhizomes have additional peaks not found in the fingerprints of leaves.

Rhizomes of all three species proved to be a rich source of proanthocyanidins. Among rhizomes of all three knotweeds the highest content of monomers and dimers was determined in Japanese knotweed, which has also proven to be the richest source of total proanthocyanidins.

## Figures and Tables

**Table 1:** Confirmation of the presence of flavan-3-ols and proanthocyanidins based on matches of the R_F_ values of standards and blue bands in tracks of STSs from rhizomes of Japanese (JK), Bohemian (BK) and giant (GK) knotweed on all HPTLC silica gel and cellulose plates. The HPTLC silica gel plate was developed with toluene–acetone–formic acid (3:6:1, *v/v*) [9], while the HPTLC cellulose plates were developed using the following developing solvents: water [17,19,20] and 1-propanol–water–acetic acid in different ratios (4:2:1, *v/v* [17,20]; 20:80:1, *v/v* [20,21,22]). The final confirmation of the compounds was only awarded to those which were detected using both stationary phases (column “Both”) and all developing solvents.

	HPTLC Stationary Phases														
	Silica Gel			Cellulose									Both		
				Developing Solvents											
	3:6:1 (*v/v*)			Water			4:2:1 (*v/v*)			20:80:1 (*v/v*)			All		
Compounds	JK	BK	GK	JK	BK	GK	JK	BK	GK	JK	BK	GK	JK	BK	GK
(+)-Catechin	+	+	+	+	+	+	+	+	+	+	+	+	+	+	+
(−)-epicatechin	+	+	+	+	+	+	+	+	+	+	+	+	+	+	+
(−)-catechin gallate	−	−	−	−	−	−	+	+	+	+	−	−	−	−	−
(−)-epicatechin gallate	+	+	+	+	+	+	+	+	+	+	+	+	+	+	+
(−)-Gallocatechin	−	−	−	+	+	+	−	−	−	−	−	−	−	−	−
(−)-Epigallocatechin	−	−	−	−	−	−	−	−	−	−	−	−	−	−	−
(−)-Gallocatechin gallate	+	+	+	−	−	−	+	+	+	+	+	+	−	−	−
(−)-Epigallocatechin gallate	−	−	−	−	−	−	−	−	−	+	+	+	−	−	−
Procyanidin B1	−	−	+	+	+	+	+	+	+	−	−	+	−	−	+
Procyanidin B2	+	+	+	+	+	+	+	+	+	+	+	+	+	+	+
Procyanidin B3	−	−	+	+	+	+	−	−	−	−	−	+	−	−	−
Procyanidin C1	+	+	+	−	+	+	+	+	+	+	+	+	−	+	+

+ Identified; − Not detected.

**Table 2:** Contents of monomers and dimers in rhizomes of Japanese, Bohemian and giant knotweed, expressed per dry mass (DM) of the plant material and quantified based on the (−)-epicatechin and procyanidin B2 calibration curves, respectively.

Knotweed	Contents of Monomers		Contents of Dimers		Contents of Monomers and Dimers	
	mg/100 g DM	kg/t DM	mg/100 g DM	kg/t DM	mg/100 g DM	kg/t DM
Japanese	299	2.99	281	2.81	580	5.80
Bohemian	152	1.52	109	1.09	261	2.61
Giant	236	2.36	217	2.17	453	4.53

**Table 3:** Comparison of our (+, − and [18]) and published data on flavan-3-ols, proanthocyanidins and their gallates identified using HPTLC–MS/MS [9,10,18] and HPLC–MS methods [7,8,12,13,14] in rhizomes and leaves of Japanese knotweed (JK), Bohemian knotweed (BK) and giant knotweed (GK). The HPTLC diol F_254S_ plates were pre-developed and developed with acetonitrile up to 9 cm.

Compound	[M-H]^−^	[M-2H]^2−^/2	[M-3H]^3−^/3	Rhizomes			Leaves		
				JK	BK	GK	JK	BK	GK
Monomers	289			[7,8,9,10,12,13,14]	+, [12,14]	+, [7,8,12,13,14]	[7,8,18]	[18]	[7,8,18]
Monomer gallates	441			[7,9,10,12,13]	+, [12]	+, [7,8,12,13]	[7,8,18]	[18]	[7,8,18]
Dimers	577			[7,8,9,10,14]	+, [14]	+, [7,8,14]	[7,8,18]	[18]	[7,8,18]
Dimer gallates	729			[9,10,13,14]	+, [14]	+, [13,14]	[7,8,18]	[18]	[7,8,18]
Dimer digallates	881			[9,10,13,14]	+, [14]	+, [13,14]	−*	[18]	[18]
Trimers	865			[9,10,14]	+, [14]	+, [14]	[18]	[18]	[18]
Trimer gallates	1017			[9,10,14]	+, [14]	+, [14]	−*	−*	[18]
Trimer digallates	1169			[14]	−, [14]	-, [14]	−*	−*	−*
Tetramers	1153			[9,10,14]	+, [14]	+, [14]	[7,8,18]	[18]	[7,8,18]
Tetramer gallates	1305			[9,10,14]	+, [14]	+, [14]	−*	−*	[18]
Pentamers	1441	720		[9,10,14]	+, [14]	+, [14]	[18]	[18]	[18]
Pentamers gallates	1593	796		[9,10]	+	+	−*	−*	[18]
Hexamers	1729	864		[9,10]	+	+	[18]	[18]	[18]
Hexamer gallates		940		[9,10]	+	+	−*	−*	[18]
Heptamers		1008		[9,10,14]	+, [14]	+, [14]	[18]	[18]	[18]
Octamers		1152	768	[9,10,14]	+, [14]	+, [14]	[18]	[18]	[18]
Nonamers		1297	863	[9,10]	+	+	[18]	[18]	[18]
Decamers		1440	960	[9,10]	+	+	[18]	[18]	[18]

+ Identified; − Not detected; −* Not detected in [18].

## Data Availability

Data are contained within the article.
